# Patient engagement to examine perceptions of perinatal depression screening with the capabilities, opportunities, motivation, and behaviors (COM-B) model

**DOI:** 10.3389/frhs.2022.845441

**Published:** 2022-09-14

**Authors:** Karen M. Tabb, Wan-Jung Hsieh, Jung Sun Sung, Tuyet Mai Ha Hoang, Megan E. Deichen Hansen, Emily Lux, Wen-Hao David Huang

**Affiliations:** ^1^School of Social Work, University of Illinois at Urbana-Champaign, Champaign, IL, United States; ^2^School of Social Work, National Taiwan University, Taipei, Taiwan; ^3^College of Education, University of Illinois at Urbana-Champaign, Champaign, IL, United States; ^4^Department of Behavioral Sciences and Social Medicine, College of Medicine, Florida State University, Tallahassee, FL, United States; ^5^Carle Foundation Hospital, Urbana, IL, United States

**Keywords:** perinatal depression, COM-B, qualitative interviews, depression screening, pregnancy, postpartum depression

## Abstract

**Background:**

Perinatal (during pregnancy and up until one year after birth) depression is one of the most common medical complications of pregnancy and is a major public health issue. The common early detection method to identify depression is to systematically administer depression screens to patients during their usual care clinic encounters. This study investigates how prenatal patients perceive depression screening and how screening informs their treatment to meet the specific needs of different racial and ethnic groups within both community and health care settings.

**Methods:**

Between June 2019 and August 2019, semi-structured in-depth interviews were conducted to explore participants' experiences of depression screening with the Edinburgh Postnatal Depression Scale (EPDS). Perinatal women (*N* = 29) consented to participate in-depth, one-on-one qualitative interviews. Trained patient-researchers (*n* = 6), women who had previously experienced a perinatal mental health problem, were trained as research team members and facilitated the interviews alongside a research assistant. All interviews were recorded and transcribed verbatim. Data was analyzed with the use of Nvivo12. Thematic network analysis was used to analyze the data.

**Results:**

Through the in-depth patient engaged qualitative interviews this study uncovered several specific motivators and behaviors related to perinatal depression screening. Using directed content analysis, several themes within a COM-B frame emerged and could be reduced to themes and further divided into two different stages: the depression screening stage and the post-screening stage.

**Conclusions:**

The results of this qualitative study provide information for health care providers to improve, adjust, and assess the process of conducting perinatal depression screening among women. The data also provide information for health care facilities to identify a better screening tool and develop and measure their screening process. These findings are essential to design comprehensive patient-centered screening protocols given the increase in state and federal policies urging universal depression screening.

## Introduction

Depression during the perinatal period is the most common complication of pregnancy and childbirth, affecting 1 in 8 women in the United States (1). Untreated perinatal depression can result in adverse outcomes for both postpartum women and their infants (2, 3). Moreover, the experience of untreated depression can be severe, resulting in suicidal ideation and/or suicide (3, 4), and maternal suicide is a leading cause of maternal mortality in the U.S. (5). Some studies report that treating depression during pregnancy is complicated by risks to the fetus (6, 7). At the same time, untreated depression during pregnancy is associated with substantial risks for adverse birth outcomes, such as low birth weight, preterm birth (2, 8), and postpartum depression (9). Given the considerable number of potential adverse physical and behavioral health outcomes for women and their offspring, it is essential to detect perinatal depression early and connect women to treatment and support. Several new models, such as psychiatric referral and consultation protocols, offer promise for assessing and treating perinatal depression (10, 11).

One promising approach to identifying perinatal depression is the practice of universal screening for depression during a health-care visit. Screening for depression during pregnancy is a clinical approach to identifying women in need of mental health diagnoses, treatment, and referrals (12, 13). It remains unclear if screening for depression during pregnancy results in better or poorer health outcomes for women and their infants (14). Currently, the United States Preventative Services Task Force (12), the American College of Obstetrics and Gynecology (15), and the Canadian Task Force on Preventive Health Care (16) have introduced screening standards in an effort to improve the detection of perinatal mental health problems. The American Psychiatric Association released a position statement on improving the quality and use of perinatal depression screening (4). The benefits of using a validated screening instrument, as well as the optimal time to screen, have not yet been determined (17). An individual's actions after completing a depression screen and motivations to seek treatment remain unknown. It also remains unclear if the act of screening results in behavior changes for the individual being screened. Few studies have involved patients in the design or delivery of research studies.

This present study is focused on the aspects of patient-engaged implementation research that includes authentic factors and conditions to scaffold intended behavioral changes resulting from the implementation of perinatal depression screening. Authentic factors, in the context of this study, represent situational factors and conditions that might facilitate or hinder the intended implementation of perinatal depression screening. In the health-care context, the Capability, Opportunity, Motivation – Behavior framework (COM-B) was proposed to articulate components and processes for intended behavioral changes of individuals upon interacting with organizations and agencies (18, 19), and can be generally interpreted as follows. *Capability* can be either physical or psychological and could be considered in terms of psychological capabilities as the result of receiving relevant training or education. *Opportunity* deals with physical and social environments within which behavioral changes occur. *Motivation* relates to perceptions, feelings, emotions, habits, and self-planning of individuals upon interacting with factors and conditions afforded by *capability* and *opportunity*. Similar to other behavioral change frameworks, the outcome of COM-B is the behavioral change of individuals within an organization or system. Therefore, this present study seeks to reveal the patient perceptions of effective depression screening processes for behavioral change purposes at the individual level in response to a system-level process.

Behavioral changes resulting from implementation of the innovation should be observed at two levels: organizational and individual. As an example, at the organizational level, incentives and obstacles could influence organizations' routines and processes for managing an individual's knowledge capital and their capabilities to make intended organizational behavioral changes (20). In other words, organizations need to absorb pertinent knowledge, skill, and ability capacities before commencing any organization-level and strategic behavioral changes. To date, few investigations have centered on people with lived experience (i.e., patients) in the discovery of motivating factors for behavioral change. For this study the COM-B framework is used to explore a qualitative research question: *What are the facilitators and barriers in depression screening and post-screening stages of mental health care for perinatal women to carry out intended behaviors?*

## Methods

### Sample

During the study period from June 2019 to August 2019, a total of 29 women consented to the study and participated in in-depth, one-on-one interviews. The participants of this study were recruited from a single public health district in Central Illinois in the context of an existing patient-centered outcome research engagement project (21–25). This study builds upon our previous work by examining what happens in the weeks to months after completing a depression screening (22). A purposive sampling approach was used in this study to better inform the research question described in the previous section. All participants provided informed consent before participation in this study. There were several inclusion criteria for participants in the study: (a) currently pregnant or within 12 months postpartum, (b) self-reported perinatal depression screening experiences, (c) 18 years of age or older, (d) English-speaking, and (e) residing in the public health district's county. Participants' eligibility was assessed through a brief telephone screening. Patients that met eligibility criteria were scheduled for individual meeting sessions followed by phone screenings. In the sample, the average age of participants was 29.5 (*SD* = 6.05). This sample was 51.7% Black (*n* = 15), 37.9% White (*n* = 11), 6.9% Asian (*n* = 2), and 3.4% multiracial (*n* = 1). More than half (55.2%) of participants had more than 12 years of formal education. On average, participants had 2.21 children (*SD* = 1.34).

### Setting

Recruitment for this interview study came from two clinic locations (one rural, one suburban) within a county public health district in the State of Illinois. The clinics provide family case management; home visits; administration of supplemental nutrition for women, infants, and children (WIC) program benefits (e.g., food vouchers for pregnant or breastfeeding women and children birth to 5 years of age); immunizations; and counseling (e.g., lactation, genetic, and nutrition) to low-income women. In order to receive most services, women must have an income <185% of the U.S. poverty line ($13,590 annual income for a single adult) and be pregnant or have children under the age of 5. In accordance with a state policy depression screening mandate, Maternal and Child Health Bureau case managers screen all pregnant women for depressive disorders using the Edinburgh Postnatal Depression Scale (EPDS) during the antenatal and postnatal periods. In addition, all outpatient and inpatient obstetric providers are mandated to perform depression screenings during pregnancy and the postpartum period in the State of Illinois.

### Procedures

An innovative patient-engaged data collection framework was adopted for the present study. This involves patient engagement at all stages of the research process from study conception through dissemination (26). The definition of a patient is a person with lived experience of a perinatal mental health disorder. The research question originated from research question–generating sessions where advisory members wanted to know if screening for depression results in behavior changes and whether patient partners might serve as researchers in interviewing other patients to learn about their experiences completing perinatal depression screenings. Six patient partners who identified as parents and had previously experienced perinatal mental health issues and/or completed depression screenings were recruited from a patient advisory board affiliated with the patient-centered outcomes research engagement projects. These patient partners served as patient consultants and attended monthly meetings on a patient-centered outcomes research advisory board. Before participating in the current study, patient partners were required to complete two qualitative interview trainings facilitated by the lead authors of this study on how to conduct human subject activities as part of the research team.

The semi-structured interview protocol in this study was developed by the advisory board members to explore participants' perceptions and experiences of receiving perinatal depression screenings and subsequent treatment decision-making. Invitations for participants were distributed by clinical staff at a partnered public health district in Illinois. Written consent was obtained from all study participants. All participants received the interview protocol at the beginning of each interview session. Participants were interviewed by the trained patient partners in English. Interviews were conducted at study participants' preferred locations, which included but were not limited to the public library's private study room, participants' homes, the clinic examination rooms of the partnered public health district, and a classroom at the local university. All interviews were conducted face-to-face, and free on-site childcare was provided to both interviewers and interviewees to promote the mother-centric nature of this study. Interviews averaged 40 minutes per participant. Each participant was compensated $50 for their participation. Data collection ended after the recruitment of 29 participants, which met a strong level of saturation in the data. Interviews were audio recorded and transcribed verbatim. All procedures were approved by the Institutional Review Board at a university in Illinois.

### Analysis

All interviews were analyzed using directed content analysis (12), which is a common method in health-care research (13), and supervised by the senior author. Our analysis process started with determining the theoretical background and categorization matrix of the COM-B framework. The coding under “motivation” follows the definitions of “reflective motivation” (increasing knowledge and understanding, eliciting feelings about behavioral targets) and “automatic motivation” (learning that induces feelings and impulses relevant to the behavioral target, imitation, habit forming, or direct influence on automatic motivational process via medication) (18). The mentioned three codes all fall under one of the motivation categories. Further, a guideline for coding rules and anchor examples was established among research team members. The research team members read all transcripts from each interview and, based on first impressions, coded text using the predetermined codes of the COM-B framework. Each interview was independently coded among research team members. To increase the rigor of the analysis, all coders cross-checked emerging themes and ratings during regular analysis meetings.

After working through 50% of the data, an intercoder reliability (ICR) test was conducted to ensure adequate ICR and establish the credibility of the findings. Coding results from two transcripts selected by the study principal investigator were used in calculating ICR and generated Cohen's kappa coefficients to verify the level of agreement between coders in qualitative text. Research indicates that the closer the kappa value is to 1, the better agreement has been reached, and values above 0.8 suggest perfect agreement (14). The Cohen's kappa values were 1.0 and 0.8 for the two selected transcripts in the study, indicating the establishment of perfect agreement among research team members.

After the re-interaction process of ICR, to further ensure quality control and enhance the trustworthiness of findings, reanalysis of existing coding was performed, which included revision of the categories and subcategories, recognition of missing texts related to the predetermined codes, and identification of newly emerged codes. Based on the finalized categorization, two coders then completed coding for all transcripts. Each step of the analysis was reviewed and organized collaboratively by the coders and the study principal investigator. The final category frequencies and interpretations based on the COM-B framework are presented in the Results section.

## Results

The results from the qualitative analysis are presented in Tables 1, 2 along with illustrative quotes corresponding to each stage. As shown in Tables 1, 2, the findings are divided into two different stages: the depression screening stage and the post-screening stage. If the patients' experiences are related to their interactions with health providers or the instruments involved in the screening stage, they are listed under the depression screening stage in Table 1. If the patients' experiences are related to resources (usually after the first screening), they are listed under the post-screening stage in Table 2. By dividing the patients' experiences into these two different phases, we can identify which parts of the screening stage influence the post-screening stage. This analysis is critical, as the screening stage either initiates or hinders patients' efforts to address depression screening outcomes, which is the intended behavior.

**Table 1:** Applying COM-B factors to patient's depression screening stage.

**Factors**	**Criteria**	**Emerging themes**	**Frequency**	**Quotes**
Capability	Psychological Comprehension of assessment and/or cognitive functioning related to the screening stage	• Language of the EPDS screening • Comprehension of what is depression screening/knowledge regarding screening	23	• “*I just feel like the questions were straight for me.”* • “*If I didn't know anything about the screen from working with pregnant women on a regular basis I probably wouldn't have understood that they were screening me for depression.”*
	Physical Physical capability to adapt to lifestyle changes related to the depression screening stage	• Unable to focus on screening	8	• “*I'm pretty sure I was given one of them (screening questionnaire) but I had a C-section and I was very drugged up on medication. Also, my kids were in the NICU…”*
Motivation	Reflective Perception of illness, belief about treatment related to the depression screening stage	• Perception of the screening tool • Results/feedback were not delivered to patients • Screening led to/did not lead to resources • Patient's perception of Self-reliance Ineffective screening questions to diagnose depression	201	• “*I think the questions are too general, too broad…I would prefer if someone would, like, talk to you one-on-one with the questionnaire.”* • “*I mean, I never got a score so it would have been nice to at least have those results…but really they didn't follow up with it at all.”* • “*When…they gave me that…the other day, I'm like, I know I need help right now. I have to mark things high.”*
	Automatic Stimuli or cure for action, mood state/disorder related to the depression screening stage	• Felt being judged by the provider • Felt being blamed by the provider • Screening caused fear	79	• “*I don't know how that's going to affect my personal life. What if I'm ever going through a custody battle with something that gets brought up…”* • “*What if you say the wrong answer maybe something may happen you have to deal with they make you go to counseling…or maybe if you have other children, they may feel like you're not stable enough to take care of them. So…I didn't know whether to answer the questions truthfully or not.”*
Opportunity	Physical Cost, access, social support, doctor and patient relationship/communication related to the depression screening stage	• Screening interval timing • Doctor-patient relationship/ communication • Screening seemed more about self-serving purpose than serving patients	82	• “*I think that there needs to be more done (screening) toward the end of the pregnancy because…the end of the pregnancy is rough.”* • “*She took more time with me, explain[ed] to me…the different things that I could do…”* • “*I think that often it can be kind of skimmed over like this…is just a part of our routine, and people don't take it as seriously as they should and don't really ask questions and take the time.”*
	Social The stigma of the disease, fear of disclosure, religious and cultural beliefs related to the depression screening stage	• Lack of privacy	31	• “*It seems like maybe it would have been more appropriate to do it individually.”*

**Table 2:** Applying COM-B to patient's post-screening stage.

**Factors**	**Criteria**	**Emerging themes**	**Frequency**	**Quotes**
Capability	Psychological Comprehension of assessment and/or cognitive functioning related to post-screening stage	• Too depressed to reach out • Stress/can't process information • Having suicidal ideation	9	• “*…like I said, when you're depressed you don't want to reach out for help…”*
	Physical Physical capability to adapt to lifestyle changes related to the post-screening stage	• Lack of physical capacity • Unable to take psychiatric medication	18	• “*I was already medicated for ADD, and I couldn't take my medications when I got pregnant or while I was breastfeeding so that kind of, like, was hard and what led to me kind of getting depressed.”* • “*Finding someone to keep my child [was a problem], or most of the time therapists are Monday through Friday. So when I was going to do it before, it was like, I have to take off work to go to the therapist. I can't take off work.”*
Motivation	Reflective Perception of illness, belief about treatment-related to post-screening stage	• Lack of mother-centric services • The resource was/was not helpful	7	• “*Nothing was really offered to me. The list of resources that they gave me I never referenced after I had initially been given that list, and I wasn't really offered anything.”* • “*I didn't really look at them [resources] …I think most of the things they gave us was more like social groups…”*
	Automatic Stimuli or cure for action, mood state/disorder related to post-screening stage	• Prefer to ask friends (trust) • Comfortability	16	• “*I was having a problem, but I don't know if I would feel comfortable necessarily just reaching out to some random...”* • “*It wasn't the resource list that motivated you to go there. It was a friend.”*
Opportunity	Physical Cost, access, social support, doctor and patient relationship/communication related to post-screening stage	• Lack of resources for fathers • Prompt and flexible process • Help from mother's support group • Lack of affordability (finance, insurance, and citizenship issue) • Lack of transportation • Prior involvement in services • Unwanted medication & treatment • Providers take initiatives to make the linkage	92	• “*I think dads are totally skipped in this process and it can affect them.”* • “*I really need to talk a little bit more, and he opens up a schedule for me. So I'm really blessed in that aspect…I can get some therapy and counseling while I'm doing my Med checks…”* • “*The first thing that was said to me…options were never given. It was pretty much like…try these pills and if that doesn't work, we'll try something else”* • “*Two months after giving birth, I don't have more Medicaid to have more insurance. So now I can't see a therapist.”* • “*It's because I'm not a US citizen, so I could have it during the pregnancy and up to 60 days after giving birth.”* • “*I feel like they probably would have followed up with me after that since I did score high then it probably would have been different.”*
	Social The stigma of the disease, fear of disclosure, religious and cultural belief related to the post-screening stage	• Stigma about perinatal depression • Experienced racism at hospital	5	• “*I really feel like I didn't get the quality care that I needed…and I felt like sometimes it was based on my race…”* • “*…my family of origin, like going to seek assistance for depression and anxiety is not something that's accepted, so I think that is something that I still hold even though I've done it on my own as an adult.”*

Emerging themes in the reflective motivation factors are patients' perceptions, critiques on the screening questionnaires, absence of the screening results, and feedback from the providers. The majority of the patients reported that the screening questionnaires were not specific enough to diagnose their depression symptoms or were not able to capture their emotions. For example, in order to draw attention to the need for help, a patient shared: “*When…they gave me that…the other day, I'm like, I know I need help right now. I have to mark things high.”* Some patients thought that having a conversation with providers would have made them feel more welcome than filling out a form. Scores and screening results were usually not delivered to patients, and as a result, not many patients were able to receive feedback and resources on the screening day. Upon closer inspection, if the patients had previous experiences with depression, they were concerned more about the quality of the screening questionnaires, while the patients who did not have previous depression were concerned more about the lack of feedback from providers.

The second significant factor at the screening stage was physical opportunity. Patients were concerned about both the time interval between screenings and the appropriate timing of the screening. As described by one patient: “*I think that there needs to be more done [screening] toward the end of the pregnancy because…the end of the pregnancy is rough*”. Many postpartum screenings were performed right after the birth (i.e., within 48 h of a woman's delivery) when women were still experiencing pain, exhaustion, and nauseousness from anesthesia. Many patients expressed that they were not able to process the questions and just wanted to “get done with it” so they could rest. Allowing a certain amount of time for women to recover and settle after birth would enhance the effectiveness of screening and increase patients' access opportunities. The quality of communication between patients and providers as well as providers' warm attention during the screening are found to be influential factors for patients. The majority of patients reported that the provider's lack of attention made them feel like the screening was just a part of a checklist rather than an opportunity.

In the patients' post-screening stage, physical opportunity was emphasized. The physical opportunity includes some factors at the system level (e.g., lack of affordability; see Table 2), which is outside of the patients' control. Most of the other factors of the patients' physical opportunities in the post-screening stage are related to their psychological capability factors during the screening. If patients lacked comprehension of screening and described lower or impaired cognitive functioning levels (e.g., feeling too stressed/depressed to process information or showing suicidal ideation), they were much more likely to either refuse or not actively seek physical opportunities. In this study, opportunities for both reflective motivation and automatic motivation could be easily lost. For example, patients who were able to understand the depression and screening stage were either already involved in the support groups or actively looked for the resources after screening, as compared to those who were in need of support but perceived too many barriers. Loss of Medicaid insurance coverage was another reported barrier to opportunity in the post-screening stage. Moreover, the options provided often did not meet the expectations and needs of the patients, as described in one interview: “*The first thing that was said to me…options were never given. It was pretty much like…try these pills and if that doesn't work, we'll try something else.”*

Another factor that influences patients' physical opportunities in the post-screening stage was the patients' prior experience with depression. If the patients had previous experiences with depression, many of them were either psychologically or physically not able to seek available resources (e.g., due to stress, inability to process information received, or inability to take psychiatric medication; see Table 2). Therefore, for them, the providers' initial approach (physical opportunity) was a critical factor in helping them adhere to the upcoming appointments and treatments. On the other hand, patients who did not have previous depression did not report notable capability factors in the post-screening stage. An example of this experience is described as follows: “*I was already medicated for ADD, and I couldn't take my medications when I got pregnant or while I was breastfeeding, so that kind of, like, was hard and what led to me kind of getting depressed.”* And in another example of capability post-screening, a patient reported: “*Finding someone to keep my child [was a problem], or most of the time therapists are Monday through Friday. So when I was going to do it before, it was like I have to take off work to go to the therapist. I can't take off work.”* These findings suggest that the patients' reflective motivation and physical opportunities in the screening stage should be designed for patients to achieve intended behaviors in the post-screening stage.

## Discussion

This study investigated how 29 perinatal patients perceived depression screenings and how screening informed their treatment while meeting the specific needs of different racial and ethnic groups within both community and health-care settings. Screening for and treating depression during pregnancy presents substantial dilemmas for both patients and stakeholders. Novel to this study, we used a patient-engagement framework where patients with lived experience and training in research methods conducted interviews with current patients (i.e., pregnant and postpartum people). As documented in past studies, for many patients, pregnancy is the first time they are assessed for depression (27, 28). As mentioned, some research shows that depression screening provides clinicians with a tool to speak with patients about depression (14, 22), but few studies examine patients' perceptions of screening and how they use the information to make informed treatment decisions (3). Thus, the findings of this study present the individual-level responses within the context of a larger system.

The versatility of COM-B in guiding health-care intervention design and pertinent implementation research is upheld in prior studies. Huang and colleagues applied the COM-B framework in their review of the literature on medication adherence (6). Their findings reported on the feasibility of COM-B in categorizing factors associated with medication adherence and non-adherence. They concluded their argument with a discussion of the advantages of the COM-B framework over existing theories of adherence. Grounded in the context of customizing a technology-enabled health communication program to promote Diabetes Prevention Program (DPP)–aligned behaviors among postpartum Latina women with recent gestational diabetes, Suri and colleagues applied COM-B to analyze focus group data (*n* = 22) provided by multiple stakeholder groups (7). Their study verified the feasibility of COM-B as a qualitative thematic analysis framework for understanding the targeted technology-enabled DPP communication program in facilitating intended behavioral changes. In our study, COM-B provides a useful framework for the implementation of perinatal depression screening as a motivating factor that either initiates or hinders patients' efforts to address treatment-seeking or related actions after completing a depression screening.

Depression screening can positively affect service use behaviors. In one study, women screened for depression subsequently used more health services for their infants compared to women who were not screened (29). In this study, to address the perceived influences, patients' reflective motivation in the screening stage can be improved in the following ways: First, the patients' perceptions about the screening can be improved through interventions such as providing patients with prior training or information regarding prenatal depression before the screening. It would be helpful in countering patients' impaired psychological capability (i.e., too depressed to reach out) and increase the automatic motivation factor in the post-screening stage (i.e., trust and comfortability). Second, the manner in which feedback/scores/resource delivery are provided on the screening day can be achieved through interventions such as clarification of the steps of this process as part of mandatory screening protocols. This can increase patients' physical opportunities in the post-screening stage (e.g., prior involvement in services, or providers taking initiatives to make the linkage), which can lead to changes in behaviors.

This study has several strengths, such as the inclusion of patients (i.e., people with lived experience) throughout each step of the study design and data collection. The inclusion of patient perspectives for meaningful engagement and to help make sense of quantitative results empowers patients with opportunities to share their voices. From this study, where former patients interviewed current patients, we learned that the physical opportunity in the screening stage can be improved in the following ways: First, improved communication between providers and patients at the screening can be achieved through interventions such as enhanced communication with providers (e.g., communicating with providers regarding how high-quality communication can prevent patients from experiencing disconnections from providers and preventive care). This could reduce patients' psychological incapability (e.g., too depressed to reach out) and increase their physical opportunities in the post-screening stage (e.g., comfortability). Second, the appropriate time frames for the screening can be achieved through interventions such as increased promotion of the available screening services and information through different media. This would increase patients' physical opportunities in the post-screening stage (e.g., prior involvement in services, or providers take initiatives to make the linkage).

This study also has some limitations. For one, the study only sought to gather the perspectives of people who completed the depression screening and did not include the perspectives of those administering the screenings. Past studies have found that providers perceive a need for more training and preparation to detect mental health needs in usual care settings (22). However, provider motivation and reasoning for their own screening practices was not included in this study and remains unknown. As another limitation, considering that the conceptualization of COM-B framework was based on nine intervention functions and seven policy categories (18), the research team adopted the COM-B framework with the intention to understand individuals' behavioral barriers induced by local organizational structure, processes, and resources. In addition, the selection of the COM-B framework allows the research team to inform organizational health-care policy development implementation with empirical evidence (30). The limitation of the COM-B framework could be its lack of granularity in deciphering study participants' experiences based on the framework's initial conceptual constructs. However, recent qualitative inquiries grounded in COM-B framework have demonstrated its applicability in studying individual behaviors in health-care contexts (31–33). Despite this limitation, COM-B provides a strong starting point to gather how depression screening relates to individual behaviors.

The abovementioned patient-centered outcome research project is considered an example of implementation research because it designed and carried out various processes and events to cultivate stakeholders' commitment to “innovation” (1) to improve the quality of mental health care for perinatal women, our primary stakeholders, in the community. The efficacy of such implementation research, however, depends on many interacting factors. In articulating the effectiveness of implementation, Klein et al. (34) proposed a process framework that consists of the climate for implementation, skills, incentive structures, obstacles, innovation-value fit, and commitment of individuals and organizations (34). In this framework, the mediating role of implementation effectiveness is essential to the ultimate effectiveness of this innovation. Taking a more dynamic perspective to articulate the fluidity of implementation research, Century et al. (35) contended that implementation research is less about the extent to which the innovation could be enacted based on its original design and intent (i.e., the fidelity of the innovation implementation) (35). It is more about understanding factors, conditions, and contexts that can influence the enactment of intended actions, namely following through with depression treatments. They identified several factors: spheres of influence, characteristics of individual end users (of the innovation), organizational and environmental factors, attributes of the innovation, implementation support strategies, and implementation over time. While the two frameworks on implementation research are decades apart, they share some common ground on organizational climate, motivations, and implementation support.

The COM-B framework in this particular study illustrates how various factors and conditions relevant to intended implementation might interact with each other at the organizational level. In another qualitative inquiry, the COM-B framework was applied to reveal facilitators and barriers for general medical practitioners to adopt a web-based telemedicine solution for depression diagnosis and patient empowerment (36). The findings suggested various time-related factors for practitioners to introduce the intervention to patients as well as overcome any difficulty in interfacing with the web-based telemedicine system. In terms of the overall reliability and validity of the COM-B framework, Keyworth et al. (37) developed and validated a 6-item questionnaire derived from the COM-B constructs (Capability, Opportunity, Motivation, and Behavior). Based on a sample of 1,387 health-care professionals working in the National Health Service in the UK, the self-evaluation questionnaire exhibited acceptable levels of reliability, content validity, discriminant validity, predictive validity, and acceptability. This study revealed some ways patients use depression screening to make informed decisions based upon beliefs of available treatment options and their motivations. Future studies are needed to further detect how health-care providers implement depression screening and decisions in the post-screening stage.

## Data availability statement

The raw data supporting the conclusions of this article will be made available by the authors, without undue reservation.

## Ethics statement

The studies involving human participants were reviewed and approved by University of Illinois at Urbana-Champaign IRB. The patients/participants provided their written informed consent to participate in this study.

## Author contributions

KT and W-HH designed the study protocol and obtained funding support. W-JH, JS, and TH transcribed the data and conducted the analysis. MDH, EL, and W-JH wrote the initial draft. All authors provided substantial contributions and reviewed the final draft.

## Funding

The authors of the study received funding from the National Institute of Minority Health Disparities (award L60 MD008481) and the Patient Centered Research Outcomes Institute (awards EA00117, 10023, and 18900). The views expressed belong to the authors rather than the funders.

## Conflict of interest

The authors declare that the research was conducted in the absence of any commercial or financial relationships that could be construed as a potential conflict of interest.

## Publisher's note

All claims expressed in this article are solely those of the authors and do not necessarily represent those of their affiliated organizations, or those of the publisher, the editors and the reviewers. Any product that may be evaluated in this article, or claim that may be made by its manufacturer, is not guaranteed or endorsed by the publisher.
