# Supplementary material for: Patient engagement to examine perceptions of perinatal depression screening with the capabilities, opportunities, motivation, and behaviors (COM-B) model
Source: Front Health Serv. 2022 Sep 14;2:845441. doi: 10.3389/frhs.2022.845441 (PMC10012820; doi:10.3389/frhs.2022.845441)
Supplement: Supplementary file 1 [file Data_Sheet_1.docx]

**Views and Perceptions of Perinatal Depression Screening across Racial/Ethnic Groups - Interview Guide**

**First of all, I would like you to tell me a little bit about yourself.**

- Please share your first name and the ages of your children?
- How long have you lived in this community?

**Now I would like you to revisit your experiences of completing perinatal screening.**

**Experience**

1. I would like you to think back to your most recent pregnancy. During that pregnancy, or shortly after you gave birth you should have received a survey about depression. **Tell me about your experience with perinatal depression screening.**

- *Setting? Provider? Clarity of questions? Ability to articulate/capture your experience?*
- *How was the timing of your screening experience? Do you think the screening times were appropriate?*
- *Did this lead to support for you and how quickly?*

**Quality**

1. After completing the screening, what feedback did you receive about your depression screening?

- *Did provider follow your process and/or give you a second screening?*

1. What feedback would you like to give about your depression screening and follow-up treatment?
2. What would you have wanted to be asked that would have addressed what was going on for you/struggles you were having?

**Resources**

1. After you were screened, if you scored high on postpartum depression scale did you feel you were able to obtain resources (i.e., support or services)?

- *Were you able to financially afford access to resources to help you alleviate symptoms? And if not what were your barriers?*

1. What were the most valuable resources or service offered to you after completing the depression screen?

**Content**

1. Was Language clear in the screening?
2. How well did you feel the screening questions captured your experience?

Those were all the questions I had for you. Again, thank you very much for all your help. **Is there anything that you would like to add or tell me?**

Lastly, do you know any other moms that you think would be interested in participating? I am looking for perinatal women and racial/ethnic minority perinatal women with experiences completing perinatal depression screening and who are age 18+ year old. This is my information in case you would like to recommend someone.
